# Synthesis, Characterization, and Biological Performances of Magnesium-Substituted Dicalcium Phosphate Anhydrous

**DOI:** 10.3390/ma17184605

**Published:** 2024-09-19

**Authors:** Jiyu Lee, Jong-Seong Bae, Yong-Il Kim, Kyung-Hyeon Yoo, Seog-Young Yoon

**Affiliations:** 1 School of Materials Science Engineering, Pusan National University, Busan 46241, Republic of Korea; jjang9135@pusan.ac.kr; 2 Busan Center, Korea Basic Science Institute, Busan 46742, Republic of Korea; jsbae@kbsi.re.kr; 3 Department of Orthodontics, Dental Research Institute, Pusan National University, Yangsan 50612, Republic of Korea; kimyongil@pusan.ac.kr; 4 JSPS Post Doc. Fellowship, Institute of Engineering Innovation, School of Engineering, The University of Tokyo, 2-11-16 Yayoi, Bunkyo-ku, Tokyo 113-8656, Japan

**Keywords:** dicalcium phosphate anhydrous, magnesium doping, in vitro test, cell test

## Abstract

Dicalcium phosphate anhydrous (DCPA, CaHPO_4_) is regarded as an orthopedic material due to its ability to match the generation of new bone to the rate of implant resorption without considering the material’s mechanical stability. Additionally, magnesium (Mg) is widely recognized for its essential function in bone metabolism, especially during the initial phases of osteogenesis. Therefore, we explored the influences of Mg ions on DCPA powder, in biological responses, and on the enhancement of osteogenic properties. Mg-DCPA powders with varying substitution levels (0, 3, 5, and 7 mol%) were produced using the co-precipitation method. In the in vitro test, precipitates began to develop on the surface of the Mg-DCPA powders after 7 days. These results indicate that Mg ions in the DCPA powder could enhance the generation of a new apatite phase when subjected to physiological fluids on the surface of the powder. In addition, the osteogenic performance of the DCPA powder was improved by adding Mg ions. The most effective magnesium substitution content in the DCPA powder in order to improve its osteogenic potential was approximately 3 mol%. Consequently, this amount of magnesium in the DCPA powder could control the maintaining time in the implantation operation to produce a new apatite phase.

## 1. Introduction

A substantial number of older people have suffered from bone fractures and related injuries annually. Therefore, there is a growing need for effective substitutes for a large number of bone repair substitute materials with good performances [1]. In recent decades, calcium phosphate compounds (CaPs) have been extensively studied as promising candidates for bone replacement surgery owing to their superior biocompatibility, their capacity to support cellular functions and gene expression, and their osteoconductive properties [2,3,4]. Of those, hydroxyapatite (HA, Ca_10_(PO_4_)_6_(OH)_2_) and tricalcium phosphate (TCP, Ca_3_PO_4_)_2_) have been the most researched phases because they have a similar chemical composition to the major inorganic component of natural bone [5,6]. The difference between these two phases is the ability to resorb in the body. While HA does not fully resorb in the body for years, TCP has better resorbability [7,8,9,10]. Despite these two CaP phases being the focus of most research, other compounds within the CaP system have remained relatively underexplored, even though they exhibit favorable resorption characteristics. Recently, there has been a growing recognition that these understudied CaP compounds offer properties comparable to TCP, particularly in orthopedic and other biomaterial applications [9,11].

Among the biodegradable CaPs, dibasic calcium phosphate anhydrous (DCPA, monetite, CaHPO_4_) and dibasic calcium phosphate dihydrate (DCPD, brushite, CaHPO_4_∙2H_2_O), which is the hydrated form of DCPA, have garnered significant interest from researchers in recent years [12,13,14]. DCPA has a continuous resorption behavior over DCPD in vivo [15], providing a proper implant resorption rate balanced with new bone formation and the ability to maintain its mechanical stability. It also shows promise for use in bone replacement surgeries because of its outstanding biocompatibility, capacity to enhance cellular functions and expressions, and osteoconductivity similar to HA and TCP [5,16,17]. Because of these benefits, DCPA is increasingly favored as an orthopedic biomaterial and is often preferred over other CaP compounds like brushite, HA, and TCP [14]. 

Research on ionic substitution in CaPs has received attention due to the wide range of external ions associated with the mineral phases in vertebrate hard tissues and the significant biological functions that many of these ions perform [18,19]. Among substituting trace element ions, magnesium (Mg) is the fourth most common cation found in the human body [20,21]. In particular, magnesium, having high biocompatibility, is found to play a crucial role in bone metabolism during the early stages of bone formation, where it encourages the proliferation of osteoblasts [22]. Due to the biological characteristics of magnesium, magnesium-substituted CaPs are receiving much attention to enhance the bioactivity of calcium phosphate-based biomaterials [23,24,25,26]. In addition, the use of DCPA for orthopedic purposes is gradually increasing over time because of the favorable properties of DCPA as a biomaterial. Therefore, incorporating magnesium into DCPA powder is a promising approach to improve bioactivity and promote bone regeneration.

The purpose of this study is to synthesize DCPA phases substituted with magnesium, a biofunctional element. The DCPA powders with different Mg amounts, determined by the (Ca + Mg)/P molar ratio, were prepared using the co-precipitation method. Then, we investigated the effect of magnesium on the structure and in vitro bioactivity of DCPA powder. In addition, a CCK-8 assay, an alkaline phosphatase (ALP) analysis, and an Alizarin Red S (ARS) analysis were conducted to investigate the interaction of hDPSCs (human dental pulp stem cells) and their osteo/odontogenic responses with DCPA and Mg-doped DCPA powders.

## 2. Materials and Methods

### 2.1. Preparation of Mg-DCPA Powders

Pure DCPA and Mg-DCPA powders were synthesized via the co-precipitation technique. As the Ca, P, Mg ions precursors, the calcium nitrate tetrahydrate (Ca(NO_3_)_2_·4H_2_O, Junsei > 98%), diammonium hydrogen phosphate ((NH_4_)_2_·HPO_4_, Junsei > 99%), and magnesium nitrate (MgNO_3_, Junsei > 99%) were used as received. To prepare the pure DCPA powder, calcium nitrate tetrahydrate (solution 1) and diammonium hydrogen phosphate (solution 2) were mixed in deionized (D.I.) water with stirring at 90 °C for 30 min in each beaker. Following the mixing of each solution, solution 2 was gradually added dropwise (13 mL/min) to solution 1 to comprise a Ca/P molar ratio of 1.0. This mixture was stirred at 90 °C for an hour, after which the precipitates were separated by vacuum filtration and then placed in an oven at 37 °C for 24 h to dry. After drying, each sample was calcined at 240 °C for 2 h (at a heating rate of 2 °C/min) and ground to fine powders. In the case of Mg-DCPA, powders with varying magnesium concentrations (3, 5, and 7 mol%) were prepared based on the assumption that magnesium ions would replace calcium ions in the DCPA lattice ((Ca +Mg)/P ratio of 1.0). Magnesium nitrate powder was dissolved in solution 1. The four samples were labeled as 0MDCPA, 3MDCPA, 5MDCPA, and 7MDCPA corresponding to pure DCPA, 3 mol% Mg, 5 mol% Mg, and 7 mol% Mg, respectively, as presented in Table 1. 

Crystalline phases of the pure DCPA and Mg-DCPA powders were identified by using a powder X-ray diffractometer (XRD, Xpert 3, PANalytical, Almelo, The Netherlands) consisting of Cu Kα radiation (λ = 1.5418 Å) produced at 40 kV and 40 mA [27]. The diffraction patterns of samples were scanned between 10° and 60°. All diffraction patterns were achieved with a scan speed of 2°/min. The infrared spectra of the pure DCPA and Mg-DCPA powders were acquired with Fourier transform infrared spectroscopy (FT-IR, Nicolet iS50 spectrometer, Thermofisher Scientific, Waltham, MA, USA). The FT-IR spectra were recorded across a range of 400 cm^−1^ to 4000 cm^−1^, with 32 scans conducted at a resolution of 4 cm^−1^ using the KBr method. In addition, the bonding structure of the Mg-DCPA powders was collected with a Raman Spectrometer (UniRAM: UniNanoTech, Giheung, Republic of Korea) with a 532 nm wavelength of laser light. The morphology of the powders was analyzed using scanning electron microscopy (SEM, Mira 3, Tesacn, Seoul, Republic of Korea) and field-emission scanning transmission electron microscopy (FE-TEM, ARM-200F, JEOL, Tokyo, Japan). The element percentages were analyzed using energy-dispersive spectroscopy (EDS in SEM, Mira 3, Tesacn, Seoul, Republic of Korea). The ability of Mg-DCPA samples to form an apatite phase was evaluated with in vitro bioactivity tests according to Kokubo’s method [28]. An amount of 1 g of the Mg-DCPA powders was immersed at 37 °C with an agitation speed of 100 rpm for 7 and 14 days in 10 mL of a simulated body fluid (SBF) solution. After being immersed in the SBF solution, all specimens were collected after being washed with deionized water and acetone.

### 2.2. Preparation of Extracts

To identify the biological performances of specimens, extracts of powders were prepared. Pure DCPA and Mg-DCPA powders were collected from the culture medium to understand the initial dissolution behavior, according to the International Standard Organization (ISO 10993-12) and previous experiments [29,30]. Depending on ISO 10993-12, the powder was soaked in α-minimum essential medium (α-MEM, Gibco, Grand Island, NY, USA) containing 10% fetal bovine serum (10% FBS, pH = 7.4) at a concentration of 0.004 g/mL and maintained at 37 °C in a 5% CO_2_ environment. Each powder has the same particle size range (45–75 µm) by sieving the powders before the biological test. All specimens were centrifuged after 24 h, and extracts of powders were achieved using a syringe filter of 0.2 mm pore size.

### 2.3. Cytotoxicity Test

To investigate the cytotoxicity of the powders, hDPSCs (PT-5025: Basel, Switzerland) at passage 7 were employed. A CCK-8 assay was conducted to evaluate the cytotoxicity of the extracts. The material extraction medium was prepared to analyze the influence of magnesium ions on the Mg-DCPA powders. All specimens were prepared using the extraction method ISO 10993-12 and according to previous experiments [29,30]. All mixture specimens with a concentration of 0.004 g/mL in the culture medium were kept in an incubator for 24 h (5% CO_2_, 37 °C atmosphere) and then filtered by a syringe filter with 0.2 µm pore size. The hDPSCs were seeded in a 48-well culture plate (2 × 10^4^ cells/well) with culture medium and placed in an incubator for 24 h before the test. The culture medium with cells was replaced with the extracted culture medium and placed in an incubator (5% CO_2_ atmosphere at 37 °C) for 1, 2, and 3 days. After 24, 48, and 72 h, a 50 µL CCK-8 solution (D-PLUSTM CCK cell viability assay kit, Dongin LS, Seoul, Republic of Korea) was added to each well and kept in an incubator for 2 h at 5% CO_2_ and 37 °C. The absorbance measurements were taken with a microplate reader (Sunrise; TECAN, Männedorf, Switzerland) at a wavelength of 450 nm following the incubation period. Three independent experiments were achieved.

### 2.4. Osteo/Odontogenic Differentiation Behavior

Osteogenic differentiation tests were performed to evaluate the influence of magnesium ions on the DCPA for hDPSCs. The process of differentiation tests was performed using the extracts according to ISO 10993-12 and previous experiments [29,30]. With this standard method, we investigated the influence of Mg ions on the cell behavior degraded from the extracts of the synthesized powder.

#### 2.4.1. Alkaline Phosphatase (ALP) Staining

The hDPSCs were cultured with an extraction medium of Mg-DCPA powders with 5 × 10^4^ cells/well in a 48-well plate. To promote differentiation, the hDPSCs were cultured in α-MEM supplemented with 10 mM β-glycerophosphate, 0.1 µM dexamethasone, and 0.1 µM ascorbic acid [30]. Then, the extracted medium was replaced at intervals of two or three days. An ALP Detection Kit (Sigma-Aldrich, St. Louis, MO, USA) was used to analyze the odontogenic differentiation of the hDPSCs at 7 and 14 days. An amount of 0.3 mL stop solution was applied to the well for 1 min, and each well was cleaned twice with D.I. water. A 0.3 mL ALP solution was applied to each well for 15 min, followed by rinsing with D.I. water. Then, the ALP substrate solution was formulated by mixing 24 mg of p-nitrophenyl phosphate (pNPP) in 5 mL of ALP buffer (0.2 M Tris-HCl, 1 mM MgCl_2_). An amount of 50 µL of the ALP substrate solution was mixed with 40 µL of cell lysate and incubated at 37 °C for 60 min. After adding 50 µL 0.5 N NaOH to stop the reaction, the absorbance data were measured at a wavelength of 405 nm.

#### 2.4.2. Alizarin Red S (ARS) Staining 

The hDPSCs were plated in the α-MEM in 48-well plates (5 × 10^4^ cells/well). The α-MEM was changed with the extracted medium after incubation for 24 h, and the extraction medium was replaced at intervals of two or three days [30]. After being cultured for 14 days, the hDPSCs were washed with phosphate-buffered saline (PBS) twice and then treated using 4% paraformaldehyde for 15 min to halt the reaction. The plates were then placed after adding 500 µL of the 2% Alizarin red solution into each well at room temperature for 30 min after the 48-well plates were washed three times with D.I. water. For statistical analysis of the ARS, 250 µL of 10% acetic acid was applied to a dry well and shaken, with the process lasting 10 min. The absorbance reading was obtained at a wavelength of 450 nm with a microplate reader (Sunrise; Tecan, Männedorf, Switzerland).

### 2.5. Statistical Analysis 

A one-way analysis of variance (ANOVA) with subsequent multiple-range tests was conducted to assess statistical significance at the 5% level among the DCPA powders. Statistical analyses were carried out using R software (version 3.5.1; R Foundation for Statistical Computing, Vienna, Austria).

## 3. Results and Discussion

### 3.1. Characterization of the Synthesized Powder

**Figure 1:**
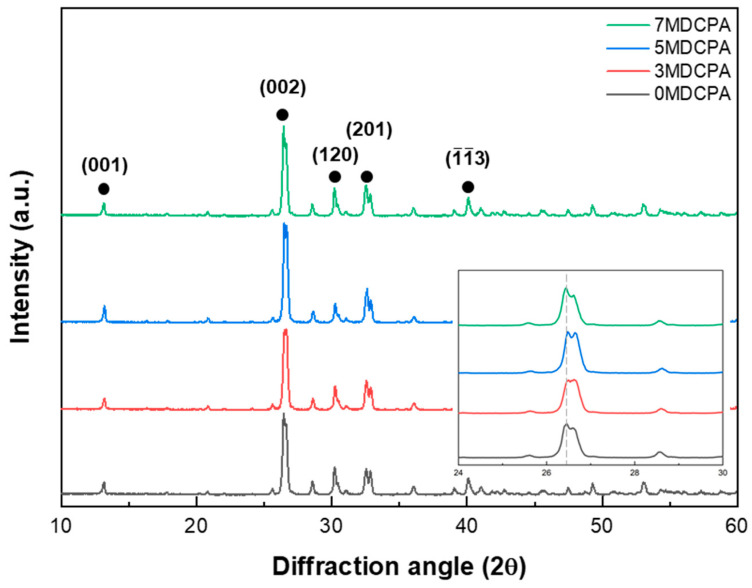
XRD patterns of the pure DCPA and Mg-DCPA powders (●: dicalcium phosphate anhydrous; DCPA).

The XRD patterns for the pure DCPA and Mg-DCPA powders with varying contents of magnesium ions are shown in Figure 1. As shown in Figure 1, all synthesized powders provided the characteristic crystalline DCPA (monetite, PDF 01-070-0360) phase [31] without any secondary phases. These results matched the previous reports; the maximum doping amount is 9 mol% for the divalent metal in calcium phosphate compounds [32]. In addition, the main characteristic peak appeared at a 2θ value of 26.46° with a related Miller index (hkl) of (002), which means the synthesized powders belong to a triclinic crystal system with space group P1 [33,34]. These results align with the structural refinements of monetite described in various studies [35,36,37]. As shown in the inside graph of Figure 1, the main characteristic peak related to the (002) plane of the DCPA phase slightly shifted to a higher 2θ angle with the addition of magnesium ions. These phenomena could indicate that Mg ions preferentially entered the DCPA phase, substituting the Ca^2+^ ion (ionic radius ~0.99 Å) with the Mg^2+^ ion (ionic radius ~0.76 Å). This substitution likely caused distortion in the crystal lattice and reduced interplanar spacing, consistent with findings reported in other studies [38,39]. Comparatively, at a higher magnesium content of 7 mol%, the main peak slightly moved to low angles. This behavior could be due to the change in the preferential site when the magnesium ion was substituted with a calcium ion.

**Figure 2:**
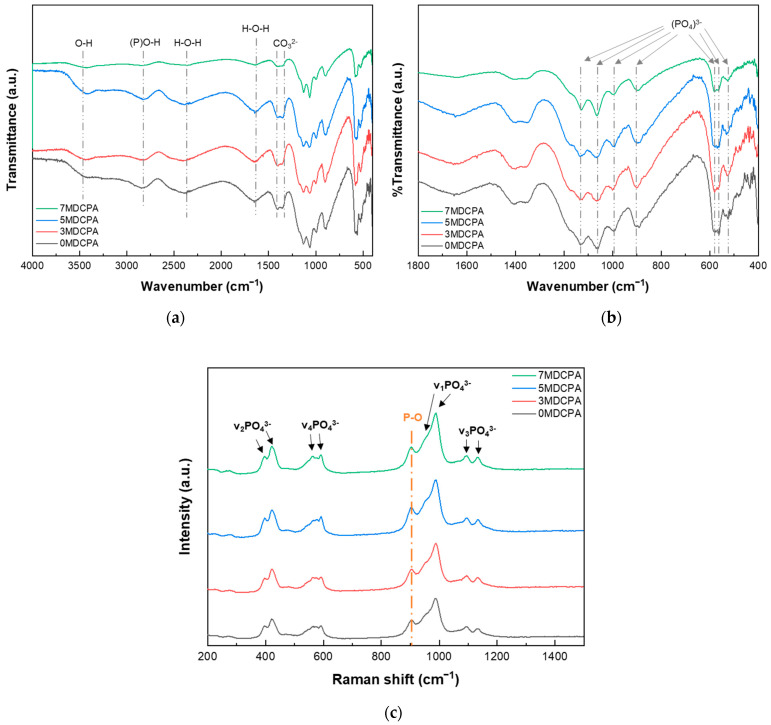
FT-IR spectra (**a**) 4000–400 cm^−1^ and (**b**) 1800–400 cm^−1^ and (**c**) Raman shift of as-synthesized pure DCPA and Mg-DCPA powders.

Figure 2a,b indicate the FT-IR spectra of the pure DCPA and Mg-DCPA powders. As shown in Figure 2a, the bands near 3480 and 1600 cm^−1^ are assigned to O-H and H-O-H stretching of adsorbed water molecules from the air, whereas the band at 2350 cm^−1^ is related to a combination of H-O-H stretching and the rotation of residual free water [40]. The band near 2800 cm^−1^ is associated with (P)O-H stretching, while the band at 905 cm^−1^ is connected to P-O(H) stretching [41], which is a unique configuration that can be seen in the DCPA phase. The bands observed in the range of 1407 to 1330 cm^−1^ are attributed to the stretching vibration of CO_3_^2−^, which results from CO_2_ adsorption. In Figure 2b, the 1180, 1140, and 1070 cm^−1^ bands are assigned to v_3_, (PO_4_)^3−^ stretching. The characteristic v_2_, (PO_4_)^3−^ stretching was observed at 480, 460 cm^−1^. Also, v_4_, (PO_4_)^3−^ and v_1_, (PO_4_)^3−^ stretching occurred at 585, 560, 525 cm^−1^, and 997 cm^−1^, respectively [42]. Figure 2c is the Raman shift of the pure DCPA and Mg-DCPA powders. In Figure 2c, the overall spectra showed principally in two sets of symmetric and asymmetric modes and two bending modes corresponding to a PO_4_^3−^-tetrahedra. A prominent band around 990 cm^−1^, associated with the tetrahedral P-O bond, shows a slight increase when the Mg ion concentration in the P-O-H+ configuration is raised [43]. As with the XRD results in Figure 1, despite the substituting of Mg ions, no newly formed bands were observed. This means that magnesium ions were stably substituted within their solubility limit when entering into the DCPA. Based on these results, such as XRD patterns, FT-IR, and Raman spectra, the synthesized powder exhibited a single phasic material, i.e., CaHPO_4_.

**Figure 3:**
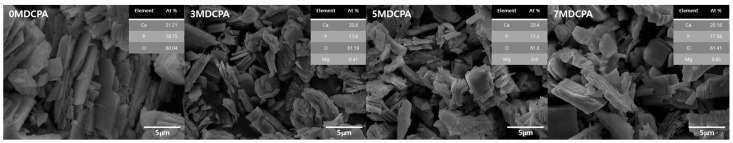
FE-SEM morphologies and atomic EDS assessments of the pure DCPA and Mg-DCPA powders.

Figure 3 illustrates the morphologies and atomic percentages of the pure DCPA and Mg-DCPA powders with different magnesium contents. The EDS results clearly show the presence of Ca, P, O, and Mg in the structure. In the case of the 0MDCPA powder, the crystals appeared to have a structure of layers of thin plates, as shown in Figure 3 [44]. On the other hand, in the presence of magnesium, the overall plate- like shapes of the DCPA were indented and changed into powders with an irregular particle structure with increasing magnesium. The particle size gradually decreased with increasing the amount of Mg up to 5 mol% and then slightly increased when the amount of magnesium ions was 7 mol%. These results could be attributed to the lattice distortion that reduces d-spacing by doping magnesium ions, as seen in Figure 1.

**Figure 4:**
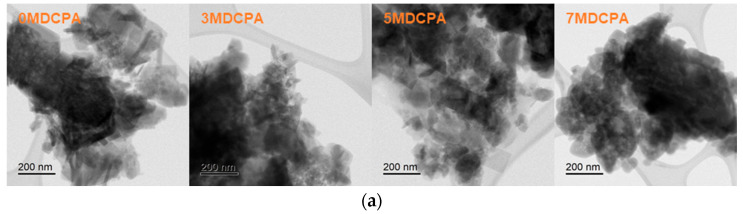

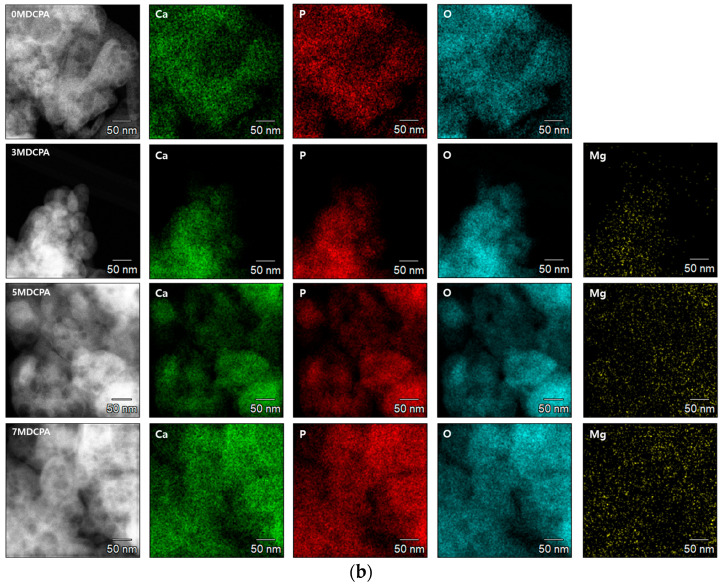
(**a**) Transmission electron microscopy (TEM) images and (**b**) EDS mapping to distribute Ca, P, O, and Mg elements of the pure DCPA and Mg-DCPA powders.

In Figure 4, TEM observations and EDS assessments were performed to further the microstructure and morphology of the pure DCPA and Mg-DCPA powders. As shown in Figure 4a, the rectangular shape of the particles inside the powder was changed to a round shape with increasing magnesium content. Figure 4b shows the HAADF and EDS mapping images of Ca, P, O, and Mg in the pure DCPA and Mg-DCPA powder samples. As shown in Figure 4b, the 0MDCPA powder had a shape with particles aggregated together; on the other hand, the Mg-DCPA powders had a shape with much smaller particles than 0MDCPA. These phenomena are caused by reducing d-spacing by substituting magnesium ions, as seen in Figure 1. As seen in the EDS results, the constituent elements such as Ca, P, O, and Mg are uniformly distributed throughout all Mg-DCPA samples. This indicates that the synthesis of both pure DCPA and Mg-DCPA powders was carried out with high consistency.

### 3.2. In Vitro Mineralization Behavior

**Figure 5:**
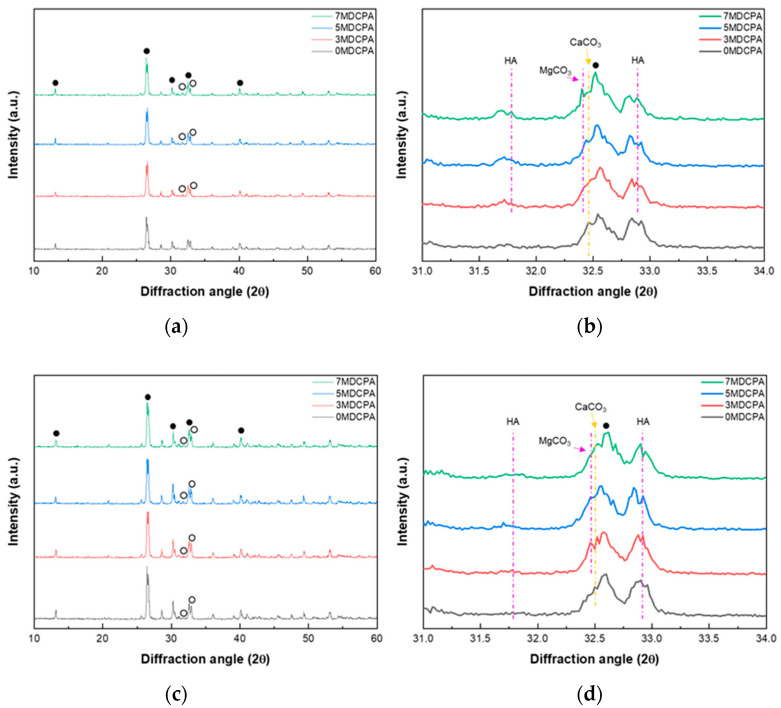
XRD patterns of pure DCPA and Mg-DCPA powders after soaking in SBF solution for (**a**,**b**) 7 and (**c**,**d**) 14 days: (**a**,**c**) 10°–60° and (**b**), (**d**) 31°–34° (●: dicalcium phosphate anhydrous; DCPA, ○: hydroxyapatite; HA).

To examine the degradation behavior of the pure DCPA and Mg-DCPA powders over immersion time, four prepared powders with varying magnesium levels were immersed in the SBF solution for an in vitro degradation. The XRD results of the pure DCPA and Mg-DCPA powders after being immersed in the SBF solution for 7 and 14 days are shown in Figure 5. When compared to before immersion, peaks in which the effect of magnesium is clearly visible are indicated with purple lines, and the rest are indicated with yellow lines. As shown in Figure 5a, new peaks around 31.78° and 32.88° corresponding to the hydroxyapatite (purple lines) phase appeared only in Mg-DCPA samples compared to the 0MDCPA sample after soaking in the SBF solution for 7 days. This suggests that the magnesium ions enhanced the biological performance of the DCPA powder in the SBF solution. In Figure 5b, the CaCO_3_ phase (yellow lines) associated with minerals was observed in all samples regardless of the amount of magnesium [45]. On the other hand, the MgCO_3_ phase (purple lines), also related to minerals, appeared only in the 5MDCPA and 7MDCPA samples. This indicates that a Mg ion was incorporated into the CaCO_3_ and formed MgCO_3_. These results show that magnesium ions contributed to mineralization of the DCPA. In Figure 5c, the HA peaks appeared in all samples, including the 0MDCPA, and increased in intensity compared to the 7-day results. The CaCO_3_ phase observed in all samples and the MgCO_3_ phase appeared in the Mg-DCPA samples regardless of magnesium content. These results mean that the mineralization is processed for 14 days, and this process more actively occurred in the Mg-DCPA samples.

**Figure 6:**
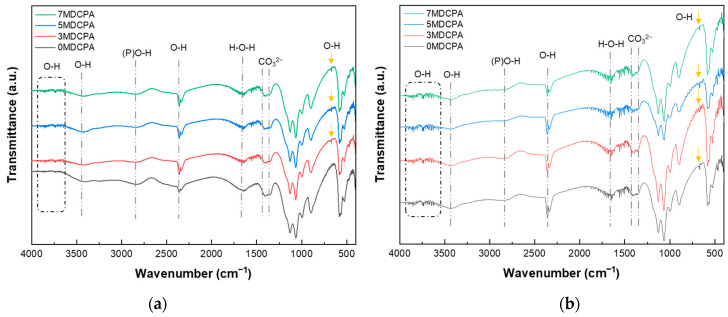
FT-IR spectra of pure DCPA and Mg-DCPA powders after soaking in SBF solution for (**a**) 7 and (**b**) 14 days.

The FT-IR spectra of the pure DCPA and Mg-DCPA powders after soaking in the SBF solution for 7 and 14 days are represented in Figure 6. Compared to the results of synthesized powder (Figure 2), new characteristic absorption bands appeared at approximately 3750, 2360, and 667 cm^−1^, corresponding to O-H vibration [46,47]. These new peaks corresponded to Ca(OH)_2_ or Mg(OH)_2_, formed during immersion in the SBF solution. The formation of a new peak at 667 cm^−1^ is known as the occurrence of DCPA degradation. As shown in Figure 6a, this peak appeared in all Mg-DCPA samples except 0MDCPA after 7 days. This means that magnesium ions could contribute to the degradation of DCPA. Furthermore, CO_3_^2−^ stretching at approximately 1407 and 1330 cm^−1^ was observed as before the in vitro test in the SBF solution. This comes from the CO_2_ adsorbed onto the surface of the DCPA powder. During immersion in the SBF solution, the adsorbed CO_2_ molecules reacted with calcium and magnesium in physiological fluid. After that, CaCO_3_ and MgCO_3_ compounds could be formed during soaking in the SBF solution. These results align with the XRD patterns shown previously in Figure 5. Figure 6b shows the FT-IR results after 14 days to further investigate the mineralization during soaking in the SBF solution. The O-H vibration peaks related to both mineralization and degradation appeared in all samples, including the 0MDCPA. Overall, in the FT-IR results, the Mg-DCPA samples show higher bioactivity compared to the 0MDCPA sample.

**Figure 7:**
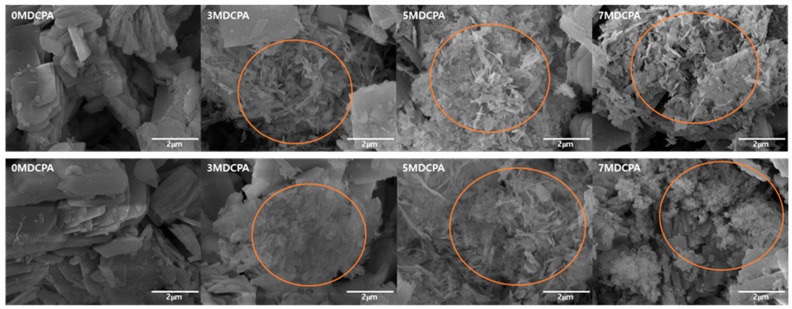
FE-SEM morphologies of pure DCPA and Mg-DCPA powders after soaking in SBF solution for 7 (**upper**) and 14 days (**lower**). Orange circle area represents the HA phase formed during the immersion test in SBF solution.

Figure 7 shows the SEM images for identifying morphology changes after SBF immersion. As can be seen in the upper images, needle-like particles began to appear on the surface of the Mg-DCPA samples following immersion in the SBF solution for 7 days. The needle-like particles might be the HA [3]. This means that the substitution of magnesium ions contributes to forming O-H bonding while immersed in an SBF solution. On the other hand, there is no significant change in particle morphologies between 7 days and 14 days in the SEM images. These results indicate that magnesium ions accelerated the mineralization of the DCPA.

### 3.3. Cytotoxicity and Osteo/Odontogenic Differentiation Behavior

**Figure 8:**
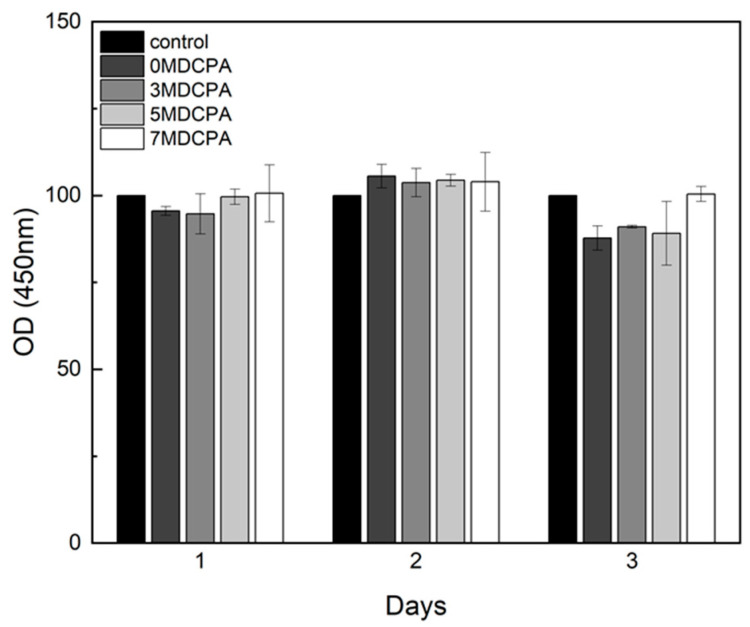
Relative cell viability of hDPSCs on the pure DCPA and Mg-DCPA powders with varying culture times at 2000 mg/well (vs. control).

The cytotoxicity and proliferation of the pure DCPA and Mg-DCPA samples were assessed with the CCK-8 assay using hDPSCs, as shown in Figure 8. Overall, all samples revealed non-cytotoxicity. At 1 day, none of the pure DCPA and Mg-DCPA showed similar values to the control. Conversely, after 2 days, the relative cell viability of all the Mg-DCPA samples exceeded that of the 0MDCPA sample, suggesting that magnesium ions could enhance the proliferation of hDPSCs [23,48]. The non-cytotoxicity result of all samples indicates that the Mg-DCPA possesses a composition suitable for clinical applications.

**Figure 9:**
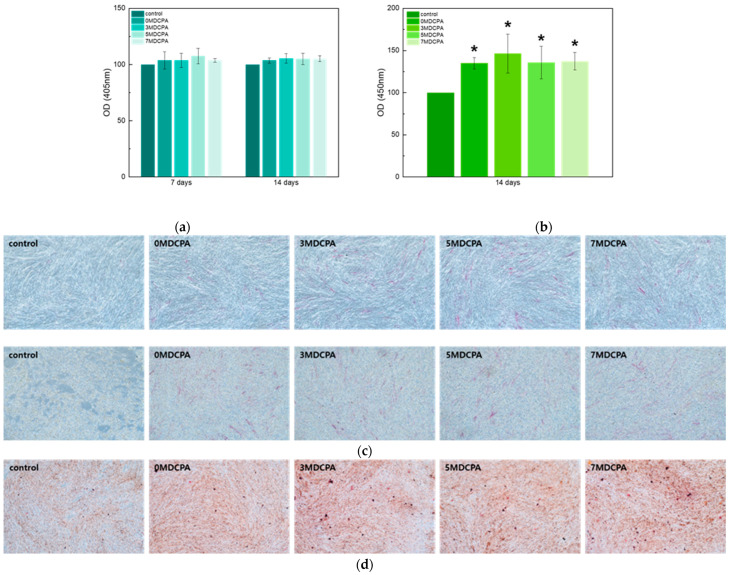
(**a**) ALP quantified results and (**b**) ARS activity results of 0.004g/mL extracted samples. (**c**) ALP staining results after 7 days (upper) and 14 days (lower) and (**d**) ARS staining results. Asterisks indicate statistical significance (* *p* < 0.05) for the control group at the corresponding time point, as assessed using an independent t-test.

ALP and ARS analyses were conducted to evaluate the osteo/odontogenic differentiation of the hDPSCs with adding magnesium ions in the DCPA powders. The ALP activity results related to the early marker of the hDPSCs placed in the extracts for the pure DCPA and Mg-DCPA samples after 7 and 14 days are shown in Figure 9. The control group was evaluated using a medium that did not undergo extraction. In Figure 9a, the cell viability of the pure DCPA and Mg-DCPA samples was slightly enhanced compared with the control after 7 days. Among them, the 5MDCPA sample has the highest fraction of differentiation, although there is an absence of a statistically significant difference among the pure DCPA and Mg-DCPA samples. These results mean that the magnesium ions support osteoblast differentiation at an early stage [49]. However, after 14 days of culture of extracts in Figure 9a, the cell viabilities did not differ much from each other and were not statistically significant. This is because the differentiation of the hDPSCs was already saturated within the 7-day culture timeframe. Meanwhile, after the 7-day culture period in Figure 9c, the extent of the stain gradually became denser with increasing the amount of magnesium.

In the ARS assay results corresponding with a late marker (Figure 9b), there was a significant increase in the mineralization of the pure DCPA and Mg-DCPA (especially the 3MDCPA) compared to the control in the ARS staining results. These assay results were analyzed with the extracts for a 14-day culture period. Regarding absorbance values, the DCPA powder samples exhibited a statistically significant increase relative to the control. This indicated that the magnesium substitution improved the osteogenic differentiation of the hDPSCs [23,48]. Furthermore, Figure 9d shows a notable difference in staining between the DCPA group and the control group. According to the biological tests, the 3MDCPA sample shows the highest potential for osteo/odontogenic differentiation. These results conclude that the magnesium ion in the DCPA powder could accelerate the differentiation of the hDPSCs and maintain their original characteristics.

## 4. Conclusions

Pure DCPA and magnesium-substituted DCPA powders were successfully synthesized through the co-precipitation method. When magnesium was introduced into the DCPA phase, d-spacing was caused by the substitution of a magnesium ion due to the difference between calcium and magnesium ionic radii. Incorporating magnesium into the DCPA phase of Mg-DCPA powder reduces the stability of the DCPA structure by causing distortions at the calcium sites, thereby increasing its reactivity. Furthermore, the presence of magnesium in the DCPA powder could enhance the accumulation of calcium- and phosphorus-containing compounds. Following 7 days in SBF, the Mg-DCPA powders began to degrade and precipitate, forming numerous needle-like crystals, which indicates the HA phase on their surface, regardless of the magnesium content. Furthermore, the CaCO_3_ and MgCO_3_ phases, implied to occur due to the mineralization of the process, were formed in all samples and Mg-DCPA samples, respectively. Conversely, when the soaking time in the SBF solution was 14 days, both the pure DCPA and Mg-DCPA powders started to form HA crystals related to needle-like crystals on the surface of the samples. Magnesium-substituted DCPA powders are anticipated to facilitate the rapid formation of a new apatite phase on their surface upon contact with physiological fluids, demonstrating a more expedited process compared to that observed with DCPA powders. After the cytotoxicity test, all samples showed non-cytotoxicity, which means that Mg-DCPA has a suitable composition for bone defect treatment. Moreover, it was observed that the biological properties of the DCPA powder were much improved with the addition of magnesium. Our experimental results indicate that the optimal magnesium substitution level in DCPA for enhancing its osteogenic potential is approximately 3 mol%. Consequently, the time required to form a new apatite phase during implantation could be modulated by adjusting the magnesium content. Following additional validation, these findings could be applied in clinical practices for the treatment of bone defects.

## Figures and Tables

**Table 1:** Notation for DCPA samples with different amounts of Mg ions.

Samples ID	Mg (mol%)
0MDCPA	0
3MDCPA	3
5MDCPA	5
7MDCPA	7

## Data Availability

The original contributions presented in the study are included in the article, and further inquiries can be directed to the corresponding author.
